# Case Report: Staged bilateral pancarpal arthrodesis using 3D-printed patient specific guides and customized hybrid plate for severe carpal joint instability associated with chronic inflammatory demyelinating polyneuropathy in a miniature dachshund

**DOI:** 10.3389/fvets.2025.1675068

**Published:** 2025-11-06

**Authors:** Adriana Franca, Isabel de Marcos Carpio, Bill Oxley, Juan J. Mínguez, Rosario Vallefuoco

**Affiliations:** 1 Pride Veterinary Referrals, Small Animal Surgery, Derby, United Kingdom; 2 Small Animal Teaching Hospital, University of Glasgow, Glasgow, United Kingdom; 3 Vet 3D, Kendal, United Kingdom; 4 Pride Veterinary Referrals, Small Animal Neurology, Derby, United Kingdom

**Keywords:** dog, chronic inflammatory demyelinating polyneuropathy, pancarpal, arthrodesis, carpal hyperextension, palmigrade

## Abstract

This clinical case report aims to describe the surgical treatment of a severe bilateral multilevel and multidirectional carpal joint instability associated with chronic inflammatory demyelinating polyneuropathy (CIDP) with staged bilateral pancarpal arthrodesis (PCA) using 3D-printed patient specific guides and customized hybrid plate in a nine-year-old female neutered Dachshund. The dog was presented due to a progressive acute onset of generalized weakness. Neurological examination, blood work, electromyography, muscle, nerve biopsies and treatment responsiveness led to a diagnosis of CIDP. Medical treatment of the CIDP resulted in resolution of muscle weakness, but did not improved the carpal hyperextension, that ultimately ended up in a severe joint instability, requiring bilateral PCA. Staged bilateral PCA using 3D-printed patient specific guides and customized hybrid plate successfully treated the carpal joint instability without associated complications and resulted in a good to excellent return to function and quality of life of the patient. PCA in chondrodystrophic and miniature breeds can be challenging. Using 3D-printed patient specific guides and customized hybrid plate in this case helped in reducing the surgical time, and offering a more stable construct.

## Introduction

Canine polyneuropathy refers to multiple nerve dysfunction and generally reflects the failure of a lower motor neuron (1). A combination of clinical signs, electrodiagnostic findings and nerve/muscle biopsy may help to confirm the diagnosis. However, the underlying cause is still seldom established (1). Multiple causes have been evocated, including immune-mediated, metabolic and degenerative conditions (1). Clinical signs of peripheral disease commonly predominate, such as generalized weakness, decreased to absent reflex activity, poor muscle tone and neurogenic muscle atrophy (1–4). In most cases, an effective treatment is not possible: some conditions may resolve spontaneously or not necessarily interfere with the patient's wellbeing, but others may impact their quality of life or even result in life-threatening complications (1, 3). The disease is usually progressive and long-term prognosis is guarded to poor due to irreversible nerve damage and associated complications (1, 5, 6). Some types of polyneuropathies have a variable and transient response to glucocorticoid therapy.

Chronic inflammatory demyelinating polyneuropathy (CIDP) is considered one of the more common neuropathies in dogs and cats (7). It is a suspected autoimmune disorder affecting mature dogs and cats (8), typically around 6–7 years of age, without sex or breed predisposition (9, 10). This condition has similarities with the neuropathy observed in humans. Symptoms usually develop gradually, following a chronic path that is often slowly progressive and sometimes relapsing. Clinical signs include weakness in all four limbs, which may progress to complete paralysis (tetraplegia), along with an unsteady gait and reduced reflexes (hyporeflexia) (7, 9). In some cases, the weakness and hypotonia leads to a palmigrady or plantigrady with muscle atrophy secondary to polyneuropathy associated denervation (11). The prognosis for CIDP is considered guarded to good, with 90% of dogs and 88% of cats responding to glucocorticoid therapy.

Common indications for pancarpal arthrodesis (PCA) may include joint damage as a result of collateral ligament injury, a hyperextension injury with or without luxation, shearing injuries, non-repairable articular fractures, severe degenerative joint disease causing pain, immune mediated arthritis or neurogenic injuries affecting only the distal limb (12, 13). Pancarpal arthrodesis in chondrodystrophic dogs can be challenging due to the breed-associated antebrachial deformities and small size of the metacarpal bones.

In this report, we describe a case of a severe bilateral carpal multilevel joint instability secondary to CIDP successfully treated with staged bilateral PCA using 3D-printed patient specific guides and customized hybrid plate.

## Case report

A nine-year-old female miniature Dachshund was referred for a progressive generalized weakness in all four limbs, worse in the pelvic limbs. Full blood profile including thyroid panel provided by the referring veterinary surgeon was largely unremarkable.

At admission, general physical examination was largely unremarkable. The orthopedic exam failed to show any abnormalities aside gait abnormalities. The neurological examination showed an ambulatory tetraparesis, more prominent on the pelvic limbs, generalized stiffness, short strided gait with hyporeflexia in all four limbs and mild muscle atrophy in the pelvic limbs. A generalized neuromuscular disorder with a symmetrical distribution such as a polyneuropathy due to a metabolic, paraneoplastic, inflammatory/infectious or degenerative etiology was highly suspected. The adrenocorticotropic hormone (ACTH) stimulation test, and neospora and toxoplasma serology tests were within normal limits and negative, respectively. A full-body computed tomography (CT) was largely unremarkable. In the following 4 weeks, the patient's stiffness and weakness progressed in all limbs, and the owners reported front paws sores, exercise reluctance, dysphonia, dysphagia and some episodes of burping (silent regurgitation). On posture evaluation, the pelvic limbs showed plantigrade stance and the thoracic limbs had a palmigrade stance with internal rotation of the carpus, causing self-trauma and pressure sores on the paws due to abnormal posture. Spinal reflexes were reduced in all limbs. Blood work including hematology and biochemistry were performed. Hematology abnormalities were non-specific and included a mild increase in the Aspartate Transferase (AST) (54.00 U/L; reference range 0–40) and Creatine Kinase (CK) (195 U/L; reference range 0–150), a mild hyperglobulinemia (54.7 g/l, reference range 26–44), mild hypoalbuminemia (21 g/l, reference range 23–36) and a mild hypocalcemia (2.26 mmol/, reference range 2.34–3.00). The C-Reactive Protein was increased (51.6 mg/L, reference range 0–10 mg/L), and the full thyroid panel values were within normal limits. Electrodiagnostic assessment revealed distinctly abnormal electromyographic activity, characterized by fibrillation potentials and positive sharp waves in multiple muscles, including the common digital extensor muscles, with more pronounced abnormalities in the pelvic limbs. Marked and consistent complex repetitive discharges were observed in the thoracic and lumbar epaxial muscles, with less prominent involvement of the cervical muscles, suggesting a chronic condition. Motor nerve conduction and supramaximal repetitive nerve stimulation were within normal limits. The F-wave study demonstrated increased minimal latency in both thoracic and pelvic limbs, indicating involvement of the ventral horn of the spinal cord or the ventral nerve root in the proximal segment, supporting a neuropathy more than a myopathy. Muscles (vastus lateralis, triceps, thoracic and lumbar epaxial muscles) and superficial peroneal nerve were biopsied for further classification of the nature and localization of the disease. The histopathology results showed a generalized myofiber atrophy and prominent fatty infiltration. Fiber type grouping was not observed, and intramuscular nerve branches appeared normal. No signs of inflammation, necrosis, organisms, or other specific cytoarchitectural abnormalities were identified. The nerve biopsy revealed a moderate reduction in nerve fiber density, with scattered myelin ovoids and nerve fibers exhibiting inappropriately thin myelin sheaths relative to axon diameter. Mild subperineurial oedema and perineurial thickening were also noted. CIDP was considered the primary differential diagnosis, as nerve histological findings in dogs with CIDP are typically mild and characterized by a variable number of fibers with abnormally thin myelin sheaths (9, 14). A course of prednisolone (initially 0.5 mg/kg per os (PO) once daily, and then increased to twice daily), L-carnitine PO (500 mg once daily), and Vitamin B complex were prescribed. Hydrotherapy was also recommended. There was a marked improvement in the pelvic limbs within 6 weeks. However, the thoracic limb palmigrady worsened, with a multilevel and multidirectional instability of both carpi, and joint effusion. Carpal orthosis was used for both legs but this resulted in the worsening of the skin pressure sores.

Because of the unresponsiveness to the corticosteroid therapy and the limited benefits of the carpal orthosis, a staged bilateral PCA was recommended. Bilateral staged procedures were performed with 8 weeks interval and a gradual reduction of steroid therapy until a dosage of 1.25 mg/kg every other day.

**Figure 1:**
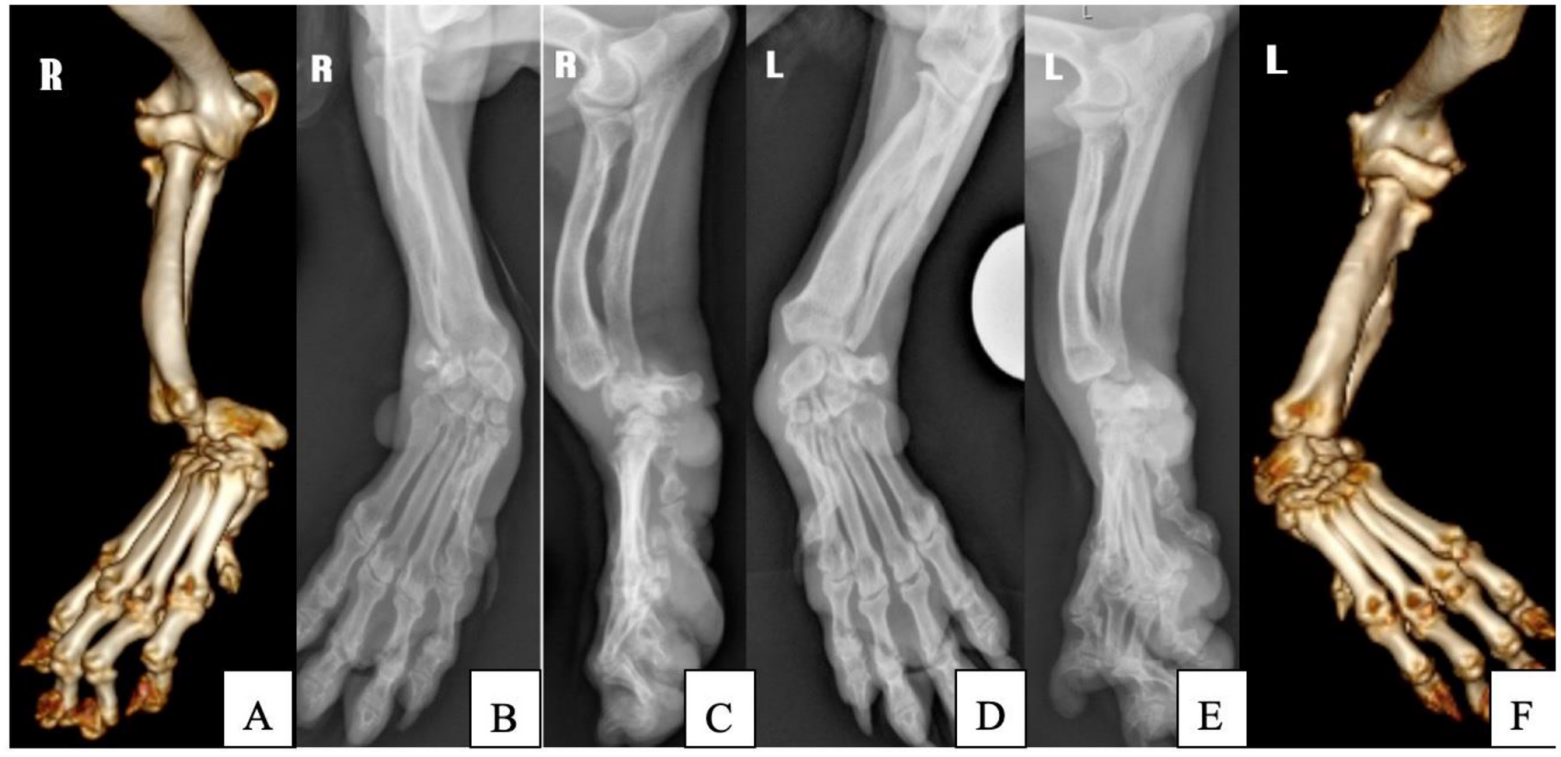
Preoperative CT 3D volume rendering and radiographic images of the right **(A–C)** and left antebrachium **(D–F)**.

The use of 3D-printed patient specific guide, and customized pancarpal arthrodesis plate was elected to reduce the surgical and anesthesia time, to allow an ideal plate-fit for the breed-associated antebrachial deformities (valgus, procurvatum and external rotation) (Figures 1A–F), in order to reduce the risk of surgical complications.

**Figure 2:**
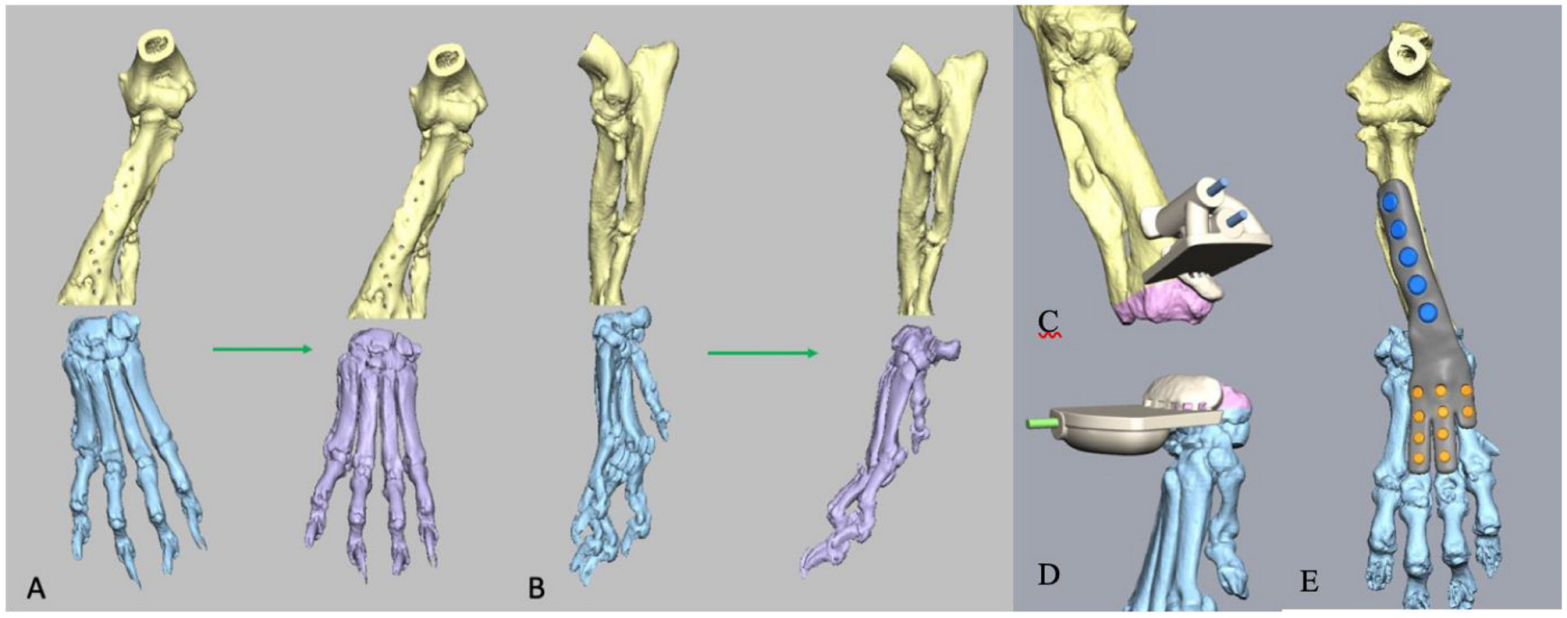
A 3D virtual representation of the antebrachia of the patient. Planned virtual osteotomies of the distal radius and carpal bones to achieve optimal antebrachio-carpal-metacarpal alignment **(A, B)**. 3D-printed patient specific guides created for the ostectomy **(C, D)**, and application of customized hybrid arthrodesis plate **(E)**.

A CT scan of both forelimbs was performed under sedation and the images were reconstructed in bone and soft tissue windows and reviewed for quantification of the antebrachial limb deformity and for surgical planning. Three-dimensional data in the form of DICOM (Digital Imaging and Communications in Medicine) files were exported to a medical image processing software (OsiriX; Pixmeo SARL, Geneva, Switzerland), and a surface-rendered representation of the forelimbs were created by using software-defined threshold settings for bone. This was exported as an STL file to CAD software (NetFabb Professional 7.2; Netfabb GmbH, Parsberg, Germany), for a 3D virtual representation of the antebrachi. The deformities could be visualized and virtual osteotomies of the distal radius and carpal bones were performed and the proximal and distal segments were reoriented with six degrees of freedom to achieve optimal antebrachio-carpal-metacarpal alignment (Figures 2A, B). Once osteotomy position and distal segment reorientation were determined, the 3D-printed patient specific guides were created for the ostectomy (Figures 2C, D). The guides included the oscillating saw guide planes, channels for 1.8 mm Ellis pins and 1.1 mm k-wires and a contact surface which precisely reflected the patient's radial and carpal bone surface cortex. Stereolithography files of the antebrachium and drill guide were exported to a software (Formlabs, Somerville, Massachusetts) associated with a 3D printer (Form 2; Formlabs) and printed by using biocompatible, autoclavable methacrylate photopolymer resin (Dental SG resin; Formlabs). The customized 316 L stainless steel plate for 2.4 mm screws was used for reduction, instead of the reduction guides, as it was designed to fit exactly with the radial and metacarpal bones cortex. The 2 distal radial holes of the plate were matching with the drilling holes of the guides and allowed the expected alignment of the bony segments at the same time (Figure 2E).

**Figure 3:**
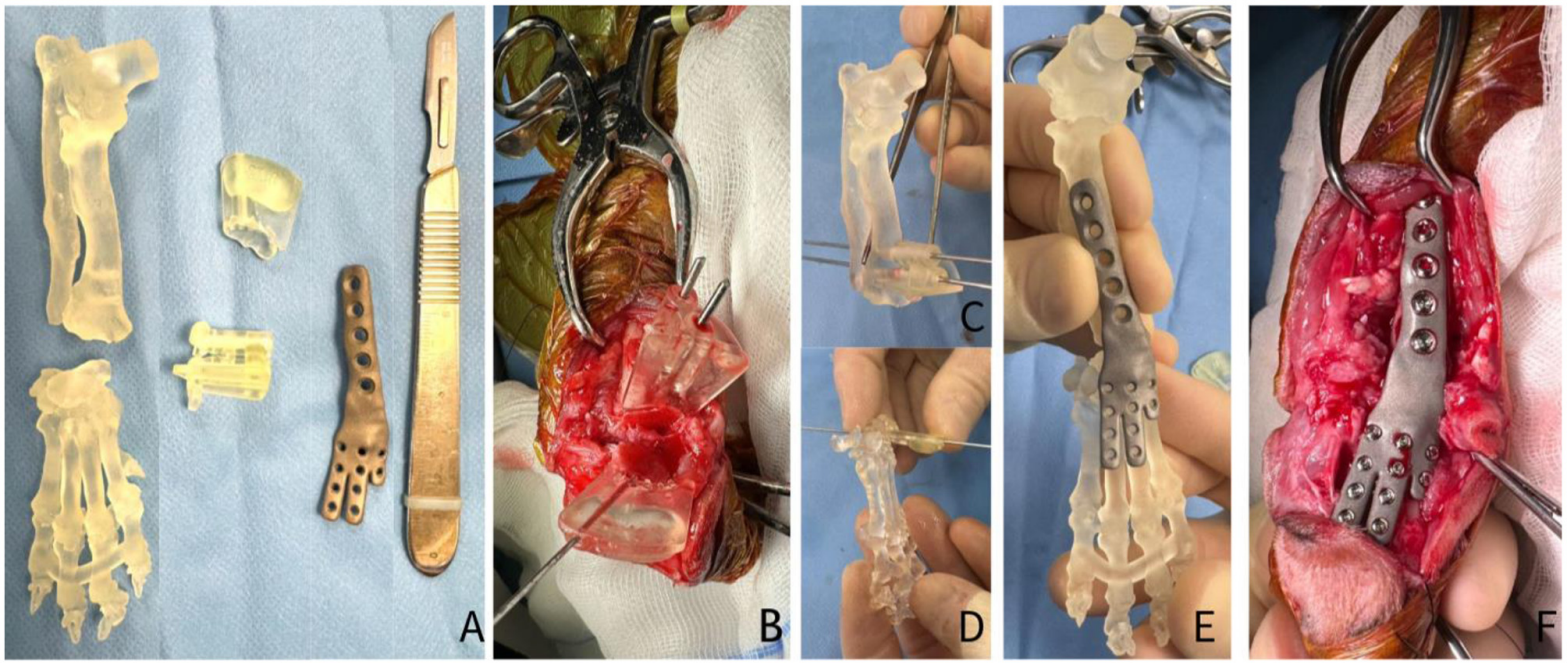
3D-printed anatomical bone models of the affected limb, patient-specific cutting guides, and a customized hybrid arthrodesis plate **(A)**. Intraoperative view showing the correct placement of the patient-specific cutting guides on the distal radius and radial carpal bone on the patient **(B)** and on the anatomical model **(C, D)**. Customized hybrid plate fitting on the antomic model, showing the exact contouring to the patient anatomy **(E)**. Intraoperative image showing the final successful placement and fixation of the customized arthrodesis plate to the carpus **(F)**.

**Figure 4:**
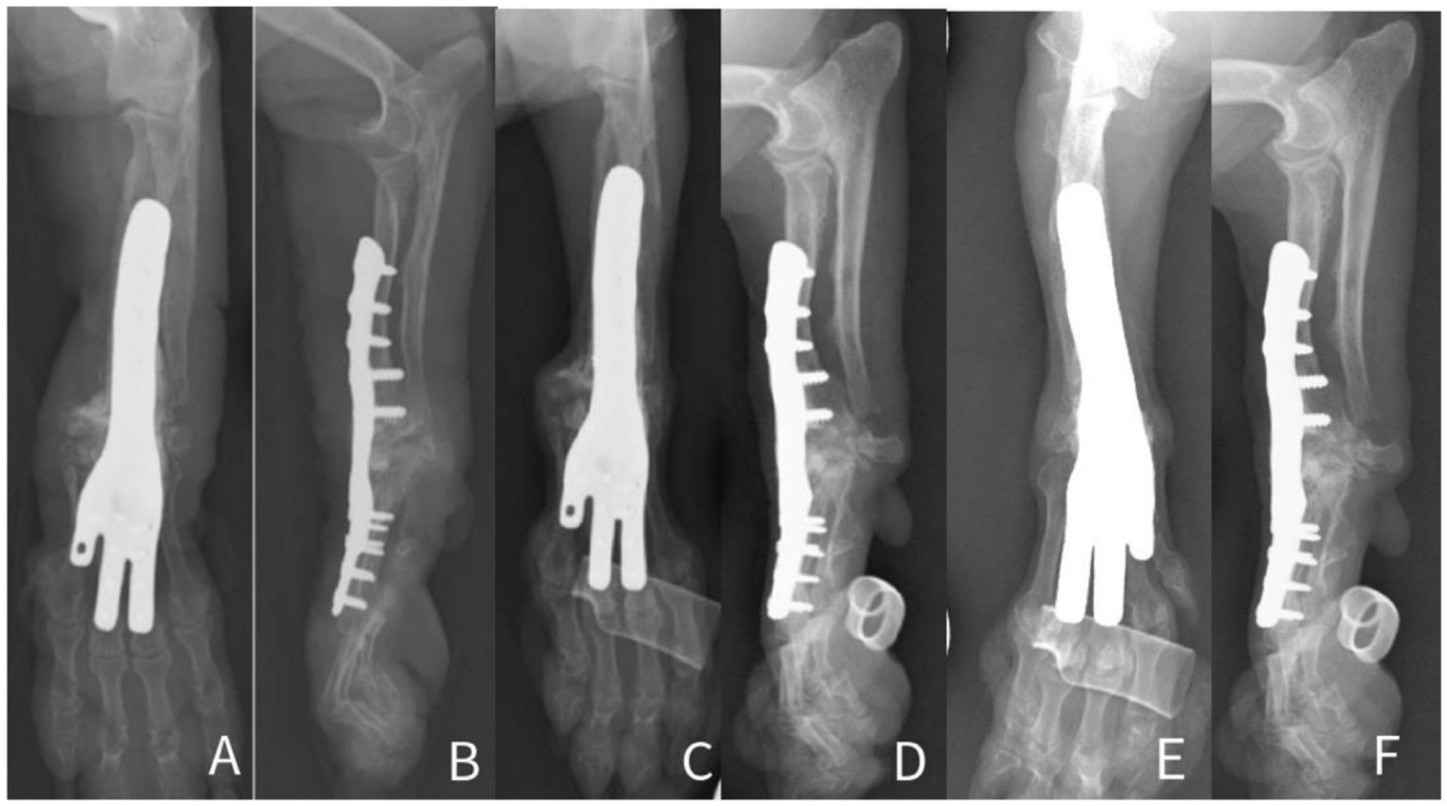
Mediolateral and caudocranial immediate **(A, B)**, 8-week **(C, D)** and 16-week **(E, F)** postoperative radiographs of the right antebrachium.

**Figure 5:**
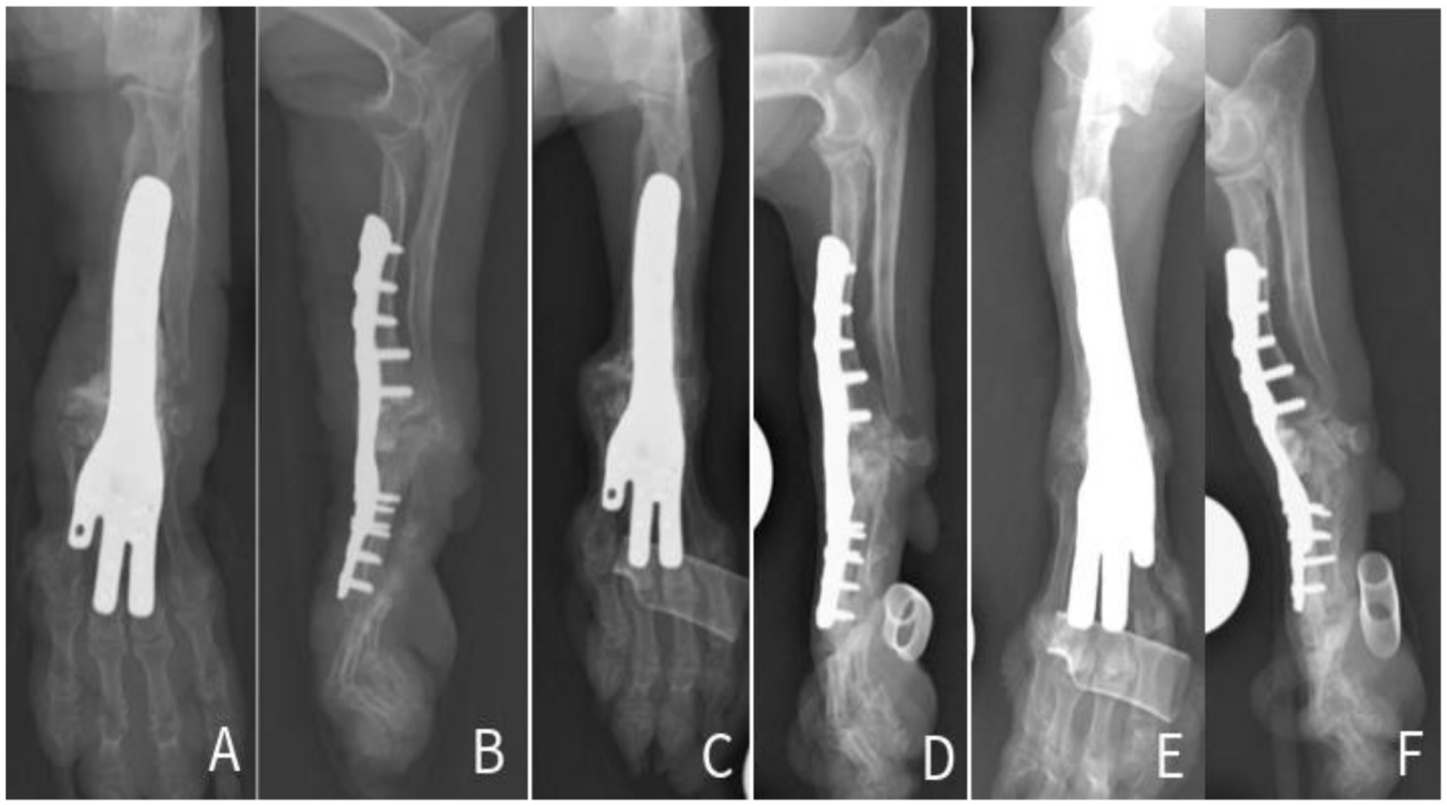
Mediolateral and caudocranial immediate **(A, B)**, 8-week **(C, D)** and 16-week **(E, F)** postoperative radiographs of the left antebrachium.

Staged procedures, starting with the right side were performed with an interval of 8 weeks. Anesthesia was performed as routine. Perioperative antibiotics (cefuroxime 20 mg/kg) were administered 60 min before the skin incision and every 90 min during the procedure. The dog was placed in lateral recumbency and the thoracic limb was prepared aseptically. A craniolateral approach to the radius extending to the distal aspect of the metacarpal-phalangeal joints was performed. A surgical guide was placed and stabilized using a 2 Ellis pins of 1.8 mm. An ostectomy of the radius was performed based on guide aid. Distal ulnar ostectomy was performed using the same approach. The ostectomy of the radiocarpal bone was performed using the sawing guide previously stabilized using a 1.1 mm k-wire. The intercarpal joint and carpal-metacarpal joint cartilage were debrided using a high-speed burr. The customized plate was applied dorsally on radius bone using 2.4 mm cortical screws and on the metacarpus II, III and IV bones using 1.5 mm cortical screws. The site was lavaged and tricalcium phosphate (Biocera-VET^®^ Bone Surgery RTU, Sheffield, United Kingdom) was applied within the inter-articular spaces (Figures 3A–F). Surgical site closure was performed in three layers as routine. Post-operative radiographs confirmed satisfactory alignment and apposition of the bony fragments and correct placement of the implants (Figures 4A, B, 5A, B).

For both surgical procedures, postoperative recovery from anesthesia was uneventful. The dog remained hospitalized for 48 h for pain management and was then discharged from the hospital with oral medication: amantadine (4 mg/kg PO once daily, 21 days), paracetamol (10 mg/kg PO three times daily, 10 days), cefalexin (22 mg/kg PO twice daily, 10 days), prednisolone (1.25 m mg/kg, every other day). A splinted dressing was applied for 4 weeks and weekly changed. Strict rest was continued for 6 more weeks. Right and left postoperative radiographs at 8 and 16 weeks (Figures 4C–F, 5C–F), showed adequate alignment of the segment, maintained position of the implants and progression of the joint fusion (Figures 4, 5). Due to a recurrence of weakness on the hindlimbs the prednisolone dose was increased to 1.25 mg/kg PO once daily.

The physiotherapy sessions were restarted and the exercise levels gradually increased to normal.

The patient was presented for a 6-month follow-up appointment. At this stage, the dog was receiving a dose of prednisolone of 1.25 mg/kg once daily. To further evaluate the progression of the mobility of the patient, a retrospective pre and postoperative validated owner questionnaire was completed (Liverpool Osteoarthritis in Dogs—LOAD). Overall assessments showed a remarkable clinical improvement and quality of life.

## Discussion

This is the first case report of a canine severe carpal instability secondary to a polyneuropathy treated with staged bilateral PCA arthrodesis using 3D-printed patient specific guides and customized hybrid plates.

A pronounced palmigrade posture associated with carpal hyperextension typically results from traumatic loss of integrity of the palmar carpal ligaments (15) and the palmar fibrocartilage (16). Also medial and lateral collateral ligaments play an important role in preventing carpal hyperextension (16). However, this abnormal stance is frequently observed also in cases of neuromuscular disorders, primarily polyneuropathies, and may also occur in dogs and cats with musculoskeletal diseases (17). Moreover, weakness, hypotonia, often leading to a palmigrady and/or plantigrady, and muscle atrophy secondary to denervation are classical motor manifestations of neuropathy (11). In human medicine, joint hypermobility observed in Ehlers-Danlos syndrome has been associated with small fiber neuropathy as one of the important pathophysiological mechanisms in addition to the connective tissue laxity (18). Equally, metatarsophalangeal joint hyperextension deformity is common in people with diabetic neuropathy (19). In dogs with chronic neuromyopathies, the loss of normal muscle strength required to support body weight results in palmigrade posture. The progressive multilevel and multidirectional carpal instability observed in this case resulted most likely from the combination of impairment of muscle strength and destruction of carpal ligaments.

Medical management with corticosteroid therapy was attempted in this dog, and was successful only to treat the weakness in the pelvic limbs, whilst the thoracic limbs progressively worsened. In dogs and cats each forelimb carries ~30% of the weight during normal stride (20–22), and this weight bearing combined with high-impact forces from running and jumping could have played a role in the carpal instability (22, 23). We can also speculate that the breed-related angular deformity of long bones in this dog and the lower body barycentre could have also been linked to a more exacerbated hyperextension instability. Initial conservative management of carpal joint laxity of this case was challenging, but after staged PCA, the patient recovered well and had an excellent return to function, with no associated complications.

PCA is generally the treatment of choice for this type of injury, when other surgical procedures or medical management fail to restore a pain-free joint function (9, 22). PCA has been considered contraindicated in patients with radial nerve injury due to the risk of self-mutilation secondary to loss of cutaneous sensation (9, 17–33). Although no sensorial deficit nor self-mutilation have been noticed in this case, cautious approach in similar cases is necessary, considering its challenging diagnosis. Complications associated with the PCA can vary from 9% to 50% (14, 24–29) and may comprise continued gait abnormality, non-union, loosening/breakage of implants, infection, metacarpal bone fractures and sclerosis of the metaphyseal metacarpal bone (16, 22, 30–32). In order to overcome the anatomical challenges of the angular deformities in this dog, to reduce the surgical time and to offer multiple screws fixation in the small metacarpal bones, 3D-printed patient specific guides a customized hybrid pancarpal arthrodesis plate were used. The accurate skeletonizing of the radius, radial carpal and metacarpal bones and the perfect fitting of the surgical guides and customized plates make this challenging surgery relatively easy to be performed. In our experience, the customized plates shortened the time of surgery, reducing the anesthesia time and potentially the risk of septic complication. Moreover, considering the small size of the metacarpal bones and consequently of the available screws, multiple distal fixation could have potentially reduced the risk of implants failure. Further *ex-vivo* and clinical studies are necessary to corroborate these hypothesis.

## Conclusion

In the presented case report, the use of staged bilateral pancarpal arthrodesis was successful in the treatment of severe multilevel and multidirectional carpal joint instability secondary to CIDP and resulted in a good to excellent return to function and quality of life of the patient without short and long-term complications. Using 3D-printed patient specific guides and customized hybrid plate could help to reduce the surgical time, and to offering a more stable construct in challenging scenario as the one described.

## Data Availability

The original contributions presented in the study are included in the article/supplementary material, further inquiries can be directed to the corresponding author.
